# Case report: “Scared to deaf”: medical-legal evaluation of a suspected post -traumatic stress disorder”

**DOI:** 10.3389/fpsyt.2024.1422002

**Published:** 2024-06-21

**Authors:** Emanuele Capasso, Mariagrazia Marisei, Marco Macculi, Pierpaolo Di Lorenzo

**Affiliations:** Department of Advanced Biomedical Sciences, University of Naples Federico II, Naples, Italy

**Keywords:** medical-legal assessment, post-traumatic stress disorder, PTSD, barotrauma, soldier, psychiatry

## Abstract

The case concerns the alleged failure by the resisting administration to fulfill obligations arising from the contract and employment relationship, as well as the violation of safety regulations leading to the workplace accident reported by an Airforce Sergeant. Following the accident, the soldier complains of developing barotraumatic hearing loss with tinnitus and reactive post-traumatic stress disorder to the traumatic event. The case provides an opportunity to analyze the relevance of forensic medical assessment and its integration with psychodiagnostic examination for the correct nosographic classification aimed at evaluating and quantifying biological damage.

## 1 Background

A number of intense stressors, including battle, natural disasters, or other events, might expose people to post-traumatic stress disorder (PTSD), a psychiatric illness (1). Sexual violence (33%) is the most common traumatic experience that can result in PTSD, followed by interpersonal-network traumatic experiences (30%) - unexpected death of a loved one, life-threatening illness of a child, and other traumatic events of a loved one and interpersonal violence (12%) (childhood physical abuse or witnessing interpersonal violence, physical assault), exposure to organized violence (3%) (refugee, kidnapped, civilian in war zone), participation in organized violence (11%) (combat exposure, witnessing death/serious injury or discovered dead bodies), and other life-threatening traumatic events (11%) (natural disaster, life-threatening motor vehicle collision, and toxic chemical exposure) (2).

According to a comprehensive study conducted in 2014, the average prevalence of PTSD in representative international population samples was 1.1%, with a range of 0.2-2.8%. Regarding the military conflict, recent research involving combat-exposed military personnel from different nations who have fought in Iraq or Afghanistan revealed that variations in PTSD rates are directly correlated with the degree of combat exposure, with an average prevalence of 6% across population samples from all services and nations (including support personnel) and 13% in infantry units exposed to combat (3–5). Twenty symptoms are categorized into four clusters within the most recent DSM-5 version of the disorder classification: intrusive and recurrent memories of the trauma, active avoidance of reminders of the event, numbness and/or negative mood changes, and changes in reactivity and arousal (6). Comorbid mental illnesses including depression and substance abuse disorders, as well as physical ailments like endocrine and autoimmune diseases, dementia, and traumatic brain damage, are frequently linked to PTSD. Twenty percent or more of those who suffer from PTSD report using alcohol or other drugs to decompress (7). The majority of individuals with PTSD are treated with a combination of drugs (such as selective serotonin reuptake inhibitors [SSRIs] or serotonin-norepinephrine reuptake inhibitors [SNRIs]) and trauma-focused psychotherapy. The choice between the two should be based on patient presentation, patient preference, and therapist expertise, according to comparative clinical trials that suggest using trauma-focused therapy with exposure or monotherapy with SSRIs that are largely comparably effective for PTSD, with some advantage to psychotherapy (8–10). Preferred treatment for people with co-occurring illnesses like depression is pharmacologic. The American Psychiatric Association (APA) and the National Institute of Mental Health (NIMH) recommend cognitive behavioral therapies, in which patients learn how to change risky behaviors—like denying oneself, re-elaborating, and experiencing one’s own emotions—and managing their anxiety and depression. In addition, the community’s and the family’s involvement and role are crucial. This case serves as a particularly pertinent example of the significance of psychodiagnostic assessment in the context of medical-legal evaluation in order to distinguish cases of PTSD from other psycho-pathological conditions, even in highly indicative cases like those related to the military environment.

## 2 Case presentation

The case presented falls under the jurisdiction of forensic medicine because it concerns the alleged failure by the resisting administration to fulfill obligations arising from the contract and employment relationship as well as the violation of safety regulations leading to the workplace accident. The present technical opinion is required for the assessment and quantification (in terms of biological disability) of the damage to the psycho-physical integrity suffered by a soldier following a workplace accident. The Air Force soldier comes to our medical-legal center for the evaluation of the damage resulting from a workplace accident in 2014, involving acoustic trauma and corneal caustication in both eyes, as well as a reactive anxious-depressive syndrome of moderate severity. The symptoms include severe bilateral tinnitus associated with hearing loss and headaches. The soldier reports suffering from insomnia and having developed post-traumatic stress disorder in response to the traumatic event. At the time of the incident, the soldier, aged 43, was serving as a Sergeant in the Air Force. On duty in a confined space, he was involved in an explosion of an electricity storage battery while replacing a faulty one located nearby. During the forensic medical evaluation, in which medical experts from both parties participated, it was deemed that the audiometric and tympanographic examinations presented in the records, also issued by qualified public institutions, were sufficient to ascertain the presence of auditory damage and could be used for its quantification. However, further investigations about the alleged psychological disturbance were necessary. The medical examiner believed that it was appropriate to proceed with a specialist consultation at the Forensic Psychotherapy Service. After outlining and agreeing upon the operational procedures for conducting the psychological assessment, the patient also gave his personal consent to proceed with video recording the interview and providing a copy to both parties. The psychological interview was conducted alongside the administration of psychiatric tests. Immediately after the explosion, the diagnosis was “caustication of the ocular surface by chemical agents, stromal corneal edema with a patch of epithelial loss in the paracentral area” and “bilateral sensorineural hearing loss more pronounced on the left”. During treatment, an anxious-depressive state with insomnia, tinnitus, hearing loss with dizziness, and reactive gastrointestinal disorders were confirmed, necessitating therapy with benzodiazepines. Two years later, during which the patient underwent numerous ophthalmic and otorhinolaryngological follow-up visits, hearing loss and dizziness persisted. Regarding the ocular injury, however, after treatment with cortisone and vasoprotective therapy, the healing process occurred with complete corneal re-epithelialization and integrity of the anterior segment of both eyes, with the optic disc and retina free from lesions.

## 3 Discussion

Regarding the auditory damage, since the immediate aftermath of the injurious event, bilateral sensorineural hearing loss at high frequencies has been documented, with a slight improvement in hearing after hyperbaric therapy sessions. The evolution of the hearing deficit at the specific frequencies established in the guidelines for assessing personal injury and widely accepted in the scientific community, as well as at higher frequencies, is presented in the following table: (**Table 1**).

**Table 1:** Evolution of hearing deficit.

Day of exam	Hearing loss (dB)						
	500 Hz	1000 Hz	2000 Hz	3000 Hz	4000 Hz	6000 Hz	8000 Hz
September 2014							
Right ear Left ear	15 30	25 45	50 60	65 70	65 75	65 90	65 100
October 2014							
Right ear Left ear	10 15	15 15	25 40	40 50	50 60	60 65	65 70
January 2015							
Right ear Left ear	10 10	15 15	35 45	45 50	50 50	50 70	55 75

The documented appearance of the hearing deficit in the immediacy of the barotrauma, together with the type of acoustic trauma experienced (bursting of a battery) and the conformational characteristic of the hearing deficit curve led to the recognition of the causal referability of the recorded hearing loss to the proven barotrauma. It seems useful to point out that in order to make a definitive judgment about the incidence of hearing loss on the subject’s validity, it is crucial to wait for an adequate amount of time, which on average consists in a few months from the occurrence of the acute event, in order to achieve stabilization of the clinical condition.

During the post-traumatic illness, the onset of tinnitus has also been documented. The specialist psycho-diagnostic observation, conducted through the technique of free conversation, integrated with clinical observation and the administration of a mental reactivity test (MMPI-2) for personality evaluation, excludes the presence of signs and symptoms compatible with a psychopathological disorder as encoded by the current nosographic manuals (DSM-5). This indicates that the certified psychological disturbance had a temporary nature, resolving after appropriate therapy. During the psychological tests, the patient demonstrated full cooperation, the provided responses appeared consistent and accurate; there didn’t seem to have been an attempt to amplify and/or conceal psychological issues. The test is deemed valid. The patient took approximately 93 minutes to complete the test, therefore cognitive functioning and abilities in attention, concentration, and comprehension seemed to be normal. The basic scales and content scales didn’t reveal the presence of psychopathological indices, but a condition of good compensation of coping abilities and subject’s adaptation. Good self-control was noted, with reflective abilities prevailing over actions. There was a generic tendency of the subject to internalize distress through shifting experiences from the emotional sphere to the somatic one, experiencing it as subjective feelings of sadness and loss, sometimes amplifying ideation characterized by worry and rumination. In conclusion, based on the conversation, observation, and testing to which the military has been subjected, the examination showed that he does not exhibit signs and symptoms compatible with a psychopathological disorder as encoded by the current nosographic manuals (DSM-5). It is noteworthy that before performing to the psycho-diagnostic evaluation In order to proceed to a psycho-diagnostic observation, the patient has been correctly and comprehensibly informed about the purpose and risks of the examination and regarding his right to refuse it in part or in full, according to Italian Law n. 219/17. After that, with regard to the same law, the patient gave his consent to proceed in written form. It is noteworthy that such a consent is ought to be expressed in written or video-recorded form and it is a legitimation of the medical act (11, 12). A solid therapeutic partnership between a doctor and patient is characterized by cooperation and a properly informed consent that is founded on adequate knowledge (13–15).

## 4 Concluding remarks

Forensic medicine, which ascertains whether a fact of a biological nature, having legal significance, is or is not related to a certain cause, sets the assessment of the causal relationship or causal nexus on the criterion of chronology, topography, efficiency, phenomenal continuity, the criterion of the exclusion of other causes. Over the years, there has been an evolution in the approach to the medico-legal problems of the psychic sphere. The relationship with the triggering event is now admitted through a rigorous assessment and in the absence of an organic substratum in psychic pathologies or in which circular causality constitutes an unavoidable element. In conclusion, even in a context usually correlated with a high incidence of PTSD, it is always and exclusively a rigorous medical-legal assessment that constitutes the element on which the diagnosis is based.

## Data availability statement

The original contributions presented in the study are included in the article/supplementary material. Further inquiries can be directed to the corresponding author.

## Ethics statement

Written informed consent was obtained from the individual(s) for the publication of any potentially identifiable images or data included in this article. Written informed consent was obtained from the participant/patient(s) for the publication of this case report.

## Author contributions

EC: Writing – review & editing, Resources, Conceptualization. MMar: Writing – original draft, Methodology. MMac: Writing – original draft, Investigation, Formal analysis. PDL: Writing – review & editing, Writing – original draft, Supervision.
